# RANTES Gene Polymorphisms Associated with HIV-1 Infections in Kenyan Population

**DOI:** 10.1155/2016/4703854

**Published:** 2016-10-16

**Authors:** Shem P. M. Mutuiri, Helen L. Kutima, Lamech M. Mwapagha, James K. Munyao, Anthony Kebira Nyamache, Irene Wanjiru, Samoel A. Khamadi

**Affiliations:** ^1^Institute of Tropical Medicine and Infectious Diseases, Jomo Kenyatta University of Agriculture and Technology, Juja, Kenya; ^2^Institute of Biotechnology, Jomo Kenyatta University of Agriculture and Technology, Juja, Kenya; ^3^Department of Plant and Microbial Sciences, Kenyatta University, Nairobi, Kenya; ^4^Center for Virus Research, Kenya Medical Research Institute, Nairobi, Kenya

## Abstract

Previous studies have reported that two single nucleotide polymorphisms (SNPs) in the RANTES gene promoter region, -403G/A and -28C/G, are associated with a slower rate of decline in CD4+ T cell count. In addition, as a ligand of the major HIV coreceptor CCR5, it is known to block HIV-CCR5 interactions in the course of the HIV infection cycle. This study was carried out with the aim of determining the occurrence of single nucleotide polymorphisms (SNPs) -403G > A and -28C > G in the promoter region of RANTES, in a subset of the Kenyan population. Genomic DNA was extracted from peripheral blood monocular cells and used to amplify the RANTES gene region. Restriction fragment length polymorphism was used to determine the genotypes of the RANTES gene. Out of 100 HIV infected individuals, 19% had G1 genotypes (403G/G, 28C/G), 30% (403A/A, 28C/C), and 50% (403G/A, 28C/C), while in healthy blood donors 13% had G4 (403G/A, 28C/C) genotypes, 22% (403A/A, 28C/C), and 54% (403G/A, 28C/C). HIV negative blood donors (54%) had higher risk of alteration to risk of HIV transmission compared to those who were HIV infected (50%). However, the risk to transmission and distribution differences was not significant (*P* = 0.092). The study showed that RANTES polymorphisms -403 and -28 alleles do exist in the Kenyan population.

## 1. Introduction

The RANTES chemokine (regulated upon activation, normal T cell expressed and secreted) is one of three natural CCR5 ligands. This chemokine interferes with the spread of HIV-1 in two mechanisms, by competitively binding to their respective receptors CCR5 thereby blocking the binding of HIV envelope glycoprotein gp120 for subsequent viral entry or by inducing internalization of the bound receptor thereby reducing the surface amounts of CCR5 [1]. Studies have shown that HIV-1-infected patients who have higher levels of RANTES in serum are partially protected against HIV-1 infection and disease progression [2, 3]. Contrarily, there is also evidence that RANTES might promote replication of some of HIV strains, particularly at high concentrations [3, 4].

It has been shown that CD4 T cells from AIDS patients produce much less RANTES than those from long-term nonprogressors [5, 6] and production of *β*-chemokines is in increased amounts, including RANTES, among HIV-exposed uninfected individuals [7, 8]. Moreover, defective CCR5 expression in CCR5 D32 homozygotes results in the downregulation of RANTES expression thus affecting the RANTES level [2, 9, 10]. The Duffy antigen receptor for chemokines (DARC) that is found on red blood cells is also associated with reduced RANTES plasma levels when it binds to chemokines including RANTES [11]. Studies have also shown that genetic variants in RANTES R1 haplotypes may downregulate RANTES gene expression and increase early HIV-1 plasma viral loads [12].

SNP studies have shown that the two polymorphisms in the promoter region of RANTES chemokine gene, 403G/A and 28C/G, and RANTES In1.1 T/C in the first intron region can influence the risk and outcome of HIV infection and diseases progression [13]. Studies have also shown that RANTES -28G allele is associated with slow rate in CD4+ depletion and inducement of RANTES expression in HIV infected individuals [14, 15]. Though this finding has not yet been confirmed among indigenous Africans, it varies across populations and ethnicities. Among the Japanese and Chinese, studies have shown that RANTES -28G frequency is lower compared to African Americans [16, 19, 20]. However, there is a clear trend towards slower rate of CD4 cell depletion in HIV-1-infected European Americans and Japanese [16]. However, reports have also shown protective effects of RANTES -28G on HIV disease progression in Thais [17] and delayed AIDS progression in HIV-1-infected Japanese haemophiliacs [18].

In RANTES, -403A variants, HIV infected individuals were reported initially among Americans with respect to 403A to progress more slowly than AIDS [16]. However in subsequent studies with large sample sizes, only RANTES -403A or RANTES In1.1 C, a RANTES intron polymorphism, was found to be associated with an increased rate of disease progression. [15, 19].

For -403G allele, previous studies have shown that it is associated with increased susceptibility to HIV infection [20] where, as in Uganda [21], North Indians [8], and Spanish populations [22], no such association of RANTES -403A/G and -28C/G polymorphism could be established in HIV-1-infected individuals. However, frequency of the -403G allele has been found to be higher in Chinese than in indigenous Africans but lower than in Caucasians, Hispanics, and African Americans [20].

Previous studies have shown that HIV-1-infected individuals have widely different rates of disease progression and several SNPs are reported to be effective in different clinical stages of HIV/AIDS patients. However, not much has been conducted among indigenous Africans. With the global and regional distribution of HIV/AIDS, protective mutation frequencies vary significantly across the globe giving each population a different genetic resistance profile to HIV infection and AIDS progression. Despite the varying responses to SNPs in relation to HIV infection, no studies have been conducted on frequencies of SNPs on RANTES gene among indigenous African populations. In this study, we evaluated the existence of the natural occurring single nucleotide polymorphisms in the RANTES gene that have been found to influence HIV disease progression and possible development of CCR5 antagonistic analogues.

## 2. Materials and Methods

### 2.1. Study Participants

After ethical approval and informed consent, 200 study subjects were enrolled in the study. These comprised of 100 HIV positive and 100 HIV-1 negative individuals during period between August and December 2007. The recruited subjects were blood donors and HIV positive patients visiting KEMRI HIV comprehensive clinic who were drug naïve and at WHO first stage of the disease. All samples were collected after obtaining l approval from KEMRI/National Ethical Review Committee (SSC 1392). All the samples collected were delinked from the participants and deidentified.

### 2.2. Laboratory Procedures

Five mL of whole blood was collected in EDTA vacutainer tubes from consenting participants who were 18 years or older. Demographic data such as age and gender, together with any history of any other disease other than HIV, were also obtained using a self-reporting questionnaire. Collected samples were tested for HIV-1 antibodies using a rapid detection kit (Determine HIV-1/2, Abbott, Japan) and Bioline HIV-1/2, Republic of Korea [23].

Peripheral blood mononuclear cells were extracted from whole blood by density gradient centrifugation using Ficoll-Paque Plus (Pharmacia) and Proviral DNA was extracted using DNAzol (Gibco BRL) and ethanol precipitation [24].

A 173 bp product of RANTES promoter was amplified by nested polymerase chain reaction (PCR) using AmpliTaq DNA polymerase (Roche Molecular Systems, Branchburg, NJ).

RANTES -28G A cytosine/guanine (C/G) transversion at position 28 in the human RANTES promoter was detected by the polymerase chain reaction-restriction fragment length polymorphism (PCR-RFLP) technique [25]. Extracted genomic DNA was amplified using the forward primer 5′′-ACA GAG ACT CGA ATT TCC GGA-3′′ and reverse primer 5′′-CCA CGT GCT GTC TTG ATC CTC-3′′ [25]. The cycling conditions for this PCR were similar, that is, one cycle at 94°C for 5 min followed by 35 cycles each of 94°C for 1 min, 62°C for 1 min, and 72°C for 1 min with a final extension step which will be carried out at 72°C for 5 min [25]. The PCR amplification was confirmed by visualization with ethidium bromide staining of the agarose gel [23].

For SNPs of RANTES, the PCR products were subjected to RFLP analysis [26, 27]. Briefly, the PCR amplicons (10 *μ*L) were digested overnight at 37°C with 0.5 *μ*L of 5000 U/mL of stock* Mnl I* (New England Biolabs, Beverly, MA, USA) and Mae III (Roche Molecular Biochemicals) in 20 *μ*L reaction volume: 2 *μ*L of 1x NE Buffer (2), 0.2 *μ*L of BSA, and 7.3 *μ*L of nuclease-free water. The digested products were separated with electrophoresis on 2% agarose gel and visualized under UV illuminator.

### 2.3. Statistical Analysis

The differences in compound genotypes and haplotypes frequencies for the RANTES promoter were presented in percentages. Fischer exact test and Pearson's Chi square were used in testing the association between HIV positive and negative status.

## 3. Results

*Mml* I and RANTES genotypes showed that the distributions of the genotypic frequencies of the RANTES polymorphism were in Hardy–Weinberg equilibrium. There were two RANTES haplotypes from the alleles (-403, -28) that were analyzed in this study and their percentage allele/genotypes frequencies corresponding to SNPs are shown in Table 1.

Three pairs of genotypes were detected: -403G/A, -28C/C; -403A/A, -28C/C; and -403G/G, -28C/G. The frequency for RANTES genotype -403G/A, -28C/C pairs was the most predominant with RANTES genotype -403G/G, -28C/G being the least. There was higher frequency in genotypes -403A/A, -28C/C, and -403G/G, -28C/G among HIV positive group against the HIV-1 negative control group. However, the predominant genotype -403G/A, -28C/C was found to be higher in the HIV-1 negative population sample as compared to HIV-1 positive although the difference was not significant (*P* = 0.092).

As shown on Table 2, HIV positive (+) subjects had RANTES -403GG, GA, and AA genotypes at a frequency of 19%, 50%, and 0%, respectively, while the HIV negative (−) subjects had RANTES -403GG, GA, and AA genotypes at a frequency of 13%, 54%, and 0%, respectively. HIV positive (+) subjects had RANTES -403GG, GA, and AA genotypes at a frequency of 19%, 50%, and 0%, respectively, while the HIV negative (−) subjects had RANTES -403GG, GA, and AA genotypes at a frequency of 13%, 54%, and 0%, respectively. From our statistical analysis, the difference of the -403GA genotype frequency between HIV positive subjects and HIV negative controls was not statistically significant, 25% for patients and 21.1% for controls (OR = 1.58, CI = 0.707–3.524, *P* = 0.2655) with a similar trend for the -28CG genotype (OR = 0.96, CI = 0.3971–2.294, *P* = 0.9172).

## 4. Discussion

Several single nucleotide polymorphisms in the RANTES gene have been reported to influence the natural course of HIV infection by regulating RANTES either up or down its activity. The most frequent of those polymorphic sites comprise -28C to G and -403G to A in the promoter region and In1.1 T/C in the first intron region of RANTES gene [28]. Usually, the two SNPs (28C/G and -403G/A) are always in complete linkage disequilibrium such that only three haplotypes are seen rather than the four that are theoretically possible [13, 16].

This was a cross-sectional study seeking to compare RANTES gene polymorphisms among HIV positive and negative individuals in Kenya. Gene polymorphisms in the -28C/G and -403G/A regions of the RANTES gene were detected. The study findings showed that the gene polymorphisms were not selected for or against as their distribution was in Hardy–Weinberg equilibrium. The study results showed that individuals with a genotype of G to G at the -403 position also had a C/G at the -28 position of the gene. Those who had a G/G at the -28 position also had an A to A at the -403 position. However, similar to the findings of Liu et al. and McDermott et al. [13, 16], no study subjects were seen to have -403G and -28G haplotypes (Table 1). This could be explained by the lack of crossover events due to the close physical proximity of the two polymorphisms and the way these single nucleotide polymorphisms have come about during human evolution. Alternatively, it could be that -403G and -28G haplotypes markedly diminish reproductive fitness [16] (Table 1).

Haplotype -403A, -28C was observed at a high frequency in both HIV-1 positive and negative subjects tested (49–55%). However, Haplotype -403G, -28C was found at somewhat lower frequency (44–50%) and with much greater variability among the tested groups. We further compared the two common genotypes frequencies in HIV negative and HIV positive individuals, G1 (-403G/A, -28C/C) and G4 (-403A/A, -28C/C), and they were found in 54% and 22% of the study subjects, respectively (Table 1). We sought to determine if HIV infection had any impact on the genotype and haplotype frequencies. However, there was no significant difference on HIV status on RANTES polymorphisms *P* = 0.092. Nevertheless, it could have implied that HIV negative blood donors (54%) had higher risk of alteration of risk of HIV transmission compared to those HIV infected (50%). However, the risk to transmission and distribution differences was not significant.

Compared to previous studies, few studies have investigated these chemokine receptor system polymorphisms in African populations. To our knowledge, only a single study had been conducted on the frequency of the RANTES -403A allele among East Africa population. However, in this study, a frequency of the RANTES −403A allele was detected in 49% of the study population [29]. These rates were similar to those among African American populations and West African blood donors (53%) [12, 16]. Consequently, these frequencies, among indigenous Africans and African Americans, were greater than those reported rates in individuals of European and Asian descent [8, 12, 16–18, 20–22, 30].

Data obtained in this study indicated that only two individuals, one from each group, had the RANTES-28G mutation that is associated with slow rate of CD4+ depletion [14–16]. Not much has been done among East Africans in relation to this mutation. Findings from this study on haplotypes in both sets of study groups were similar to those obtained from previous studies among indigenous African population and ancestral haplotype inheritance being sighted as the reason [19]. RANTES (CCL5) is one important chemokine in diseases inflammation and modulates over 100 diseases. Besides its physiological role as a chemokine, RANTES, a natural ligand of CCR5, is a potent HIV-1 inhibitor [1]. Therefore, there are a lot of efforts worldwide in pursuing it to engineer RANTES derivatives with high anti-HIV-1 potency to use it as microbicides or a CCR5 antagonist without activating CCR5 [31].

Liu et al. [13] previously identified the single nucleotide polymorphism sites, -28C/G and -403G/A, in the promoter region of RANTES and demonstrated that this resulted in increased transcriptional activity and subsequent expression of RANTES in HIV infected individuals thereby delaying disease progression.

Our study confirms similar trends of distribution and lack of association of RANTES -403 and -28 genotypes with higher transmission rates [28].

The findings of this study have very important implications for studies designed to test vaccines or therapeutic agents. Although countries in sub-Saharan Africa like Kenya may well prove to be suitable populations for vaccine trials, it is important to confirm the distribution of genetic variations in RANTES in the populations, even though it has been found to be extremely heterogeneous. The major limitation of this study was that it was cross-sectional and also involved a very small sample size that was not representative of the Kenyan population.

## 5. Limitations of the Study

The study did not include other RANTES SNPs that have been associated with higher disease transmission risk like the RANTES In1.1 T allele [28].

In addition, the study utilized low number of study subjects for both the HIV positive case subjects and HIV negative control blood donors. However, it should be noted that the study was undertaken as the first genetic survey on RANTES gene polymorphisms on a Kenyan population and as such there were no previous studies on the distribution to advice on the sample size at the time of conducting the study. A larger sample size and study are required to rule out the association of RANTES -403 and -28 genotypes with higher HIV transmission rates in the Kenyan population.

## 6. Conclusions

RANTES polymorphisms in -403 and -28 alleles do exist in Kenya. The data from this study showed no significant difference in distribution of RANTES polymorphisms in both HIV positive and negative study participants. However, there is need to conduct prospective studies with large sample sizes to get a clearer picture on this. In addition, with RANTES being critical chemokine and competitively inhibitor for HIV-1 by binding to its receptor CCR5, there is need for further exploration for CCR5 antagonist, which could also be a potential anti-HIV agent.

## Figures and Tables

**Table 1:** RANTES promoter compound genotypes and haplotypes frequencies (%) in HIV positive and negative subjects. Numbers in parenthesis give data in percentages.

		RANTES promoter SITE		Total	HIV-1 +ve	HIV-1 −ve
		-403	-28	*n* = 200 (%)	*n* = 100	*n* = 100
Genotype	1	G/G	C/G	16 (8)	19	13
	2	A/A	C/C	26 (13)	30	22
	3	A/A	G/G	0	0	0
	4	G/A	C/C	52 (26)	50	54
	5	G/A	G/C	0	0	0
	6	A/A	G/C	1 (0.5)	1	1

Haplotype	I	A	C	52 (26)	55	49
	II	G	C	47 (23.5)	44	50
	III	A	G	1	1	1

**Table 2:** Association of RANTES promoter compound genotypes and haplotypes frequencies (%) with HIV infection.

RANTES promoter	HIV-1 +ve *n* = 100 (same as %)	HIV-1 −ve *n* = 100 (same as %)	Odds ratio	95% confidence interval (CI)	*P* value
-403G/A genotype					
G/G	19	13	1.58	0.707–3.524	**0.2655**
G/A	50	54			
AA	0	0			

-28C/G genotype					
CC	30	22	0.96	0.3971–2.294	**0.9172**
CG	20	14			
GG	0	0			

## References

[1] Cocchi F., DeVico A. L., Garzino-Demo A., Arya S. K., Gallo R. C., Lusso P. (1995). Identification of RANTES, MIP-1*α*, and MIP-1*β* as the major HIV-suppressive factors produced by CD8^+^ T cells. *Science*.

[2] Paxton W. A., Neumann A. U., Kang S. (2001). RANTES production from CD4^+^ lymphocytes correlates with host genotype and rates of human immunodeficiency virus type 1 disease progression. *Journal of Infectious Diseases*.

[3] Kelly M. D., Naif H. M., Adams S. L., Cunningham A. L., Lloyd A. R. (1998). Cutting edge: dichotomous effects of *β*-chemokines on HIV replication in monocytes and monocyte-derived macrophages. *Journal of Immunology*.

[4] Kinter A., Catanzaro A., Monaco J. (1998). CC-chemokines enhance the replication of T-tropic strains of HIV-1 in CD4^+^ T cells: role of signal transduction. *Proceedings of the National Academy of Sciences of the United States of America*.

[5] Saha K., Bentsman G., Chess L., Volsky D. J. (1998). Endogenous production of *β*-chemokines by CD4^+^, but not CD8^+^ T-cell clones correlates with the clinical state of human immunodeficiency virus type 1 (HIV-1)-infected individuals and may be responsible for blocking infection with non-syncytium-inducing HIV-1 in vitro. *Journal of Virology*.

[6] Paxton W. A., Kang S., Liu R. (1999). HIV-1 infectability of CD4^+^ lymphocytes with relation to *β*-chemokines and the CCR5 coreceptor. *Immunology Letters*.

[7] Paxton W. A., Martin S. R., Tse D. (1996). Relative resistance to HIV-1 infection of CD4 lymphocytes from persons who remain uninfected despite multiple high-risk sexual exposures. *Nature Medicine*.

[8] Suresh P., Wanchu A., Bhatnagar A., Sachdeva R. K., Sharma M. (2007). Spontaneous and antigen-induced chemokine production in exposed but uninfected partners of HIV type 1-infected individuals in North India. *AIDS Research and Human Retroviruses*.

[9] Clegg A. O., Ashton L. J., Biti R. A. (2000). CCR5 promoter polymorphisms, CCR5 59029A and CCR5 59353C, are under represented in HIV-1-infected long-term non-progressors. *AIDS*.

[10] Samson M., Libert F., Doranz B. J. (1996). Resistance to HIV-1 infection in caucasian individuals bearing mutant alleles of the CCR-5 chemokine receptor gene. *Nature*.

[11] He W., Neil S., Kulkarni H. (2008). Duffy antigen receptor for chemokines mediates trans-infection of HIV-1 from red blood cells to target cells and affects HIV-AIDS susceptibility. *Cell Host and Microbe*.

[12] Duggal P., Winkler C. A., An P. (2005). The effect of RANTES chemokine genetic variants on early HIV-1 plasma RNA among African American injection drug users. *Journal of Acquired Immune Deficiency Syndromes*.

[13] Liu H., Shioda T., Nagai Y. (1999). Distribution of HIV-1 disease modifying regulated on activation normal T cell expressed and secreted haplotypes in Asian, African and Caucasian individuals. French ALT and IMMUNOCO Study Group. *AIDS*.

[14] Liu H., Chao D., Nakayama E. E. (1999). Polymorphism in RANTES chemokine promoter affects HIV-1 disease progression. *Proceedings of the National Academy of Sciences of the United States of America*.

[15] An P., Nelson G. W., Wang L. (2002). Modulating influence on HIV/AIDS by interacting RANTES gene variants. *Proceedings of the National Academy of Sciences of the United States of America*.

[16] McDermott D. H., Beecroft M. J., Kleeberger C. A. (2000). Chemokine RANTES promoter polymorphism affects risk of both HIV infection and disease progression in the Multicenter AIDS Cohort Study. *AIDS*.

[17] Wichukchinda N., Nakayama E. E., Rojanawiwat A. (2006). Protective effects of IL4-589T and RANTES-28G on HIV-1 disease progression in infected Thai females. *AIDS*.

[18] Koizumi Y., Kageyama S., Fujiyama Y. (2007). RANTES 28G delays and DCSIGN 139C enhances AIDS progression in HIV type 1-infected Japanese hemophiliacs. *AIDS Research and Human Retroviruses*.

[19] Gonzalez E., Dhanda R., Bamshad M. (2001). Global survey of genetic variation in CCR5, RANTES, and MIP-1*α*: impact on the epidemiology of the HIV-1 pandemic. *Proceedings of the National Academy of Sciences of the United States of America*.

[20] Zhao X.-Y., Lee S. S., Wong K. H. (2004). Effects of single nucleotide polymorphisms in the RANTES promoter region in healthy and HIV-infected indigenous Chinese. *European Journal of Immunogenetics*.

[21] Cooke G. S., Tosh K., Ramaley P. A. (2006). A polymorphism that reduces RANTES expression is associated with protection from death in HIV-seropositive Ugandans with advanced disease. *Journal of Infectious Diseases*.

[22] Vidal F., Peraire J., Domingo P. (2006). Polymorphism of RANTES chemokine gene promoter is not associated with long-term nonprogressive HIV-1 infection of more than 16 years. *Journal of Acquired Immune Deficiency Syndromes*.

[23] Nyamache A. K., Muigai A. W. T., Nganga Z., Khamadi S. A. (2012). HIV type 1 genetic diversity and naturally occurring polymorphisms in HIV type 1 Kenyan isolates: implications for integrase inhibitors. *AIDS Research and Human Retroviruses*.

[24] Khamadi S. A., Ochieng W., Lihana R. W. (2005). HIV type 1 subtypes in circulation in northern Kenya. *AIDS Research and Human Retroviruses*.

[25] Kameyoshi Y., Dörschner A., Mallet A. I., Christophers E., Schröder J.-M. (1992). Cytokine RANTES released by thrombin-stimulated platelets is a potent attractant for human eosinophils. *Journal of Experimental Medicine*.

[26] Al Hajeer A. H., Al Sharif F., Ollier W. E. R. (1999). A polymorphism at position −403 in the human RANTES promoter. *European Journal of Immunogenetics*.

[27] Ai Sharif F., Ollier W. E. R., Hajeer A. H. (1999). A rare polymorphism at position −28 in the human RANTES promoter. *European Journal of Immunogenetics*.

[28] Rathore A., Chatterjee A., Sivarama P., Yamamoto N., Singhal P. K., Dhole T. N. (2008). Association of RANTES −403 G/A, −28 C/G and In1.1 T/C polymorphism with HIV-1 transmission and progression among North Indians. *Journal of Medical Virology*.

[29] Katz D. A., John-Stewart G. C., Richardson B. A. (2010). CCR5, RANTES and SDF-1 polymorphisms and mother-to-child HIV-1 transmission. *International Journal of Immunogenetics*.

[30] Fernandez R. M., Borrego S., Marcos I., Rubio A., Lissen E., Antinolo G. (2003). Fluorescence resonance energy transfer analysis of the RANTES polymorphisms −403G → A and −28G → C: evaluation of both variants as susceptibility factors to HIV type 1 infection in the Spanish population. *AIDS Research and Human Retroviruses*.

[31] Vangelista L., Secchi M., Lusso P. (2008). Rational design of novel HIV-1 entry inhibitors by RANTES engineering. *Vaccine*.

