# Corrigendum to “RANTES Gene Polymorphisms Associated with HIV-1 Infections in Kenyan Population”

**DOI:** 10.1155/2017/6230176

**Published:** 2017-10-25

**Authors:** Shem P. M. Mutuiri, Helen L. Kutima, Lamech M. Mwapagha, James K. Munyao, Anthony Nyamache Kebira, Irene Wanjiru, Samoel A. Khamadi

**Affiliations:** ^1^Institute of Tropical Medicine and Infectious Diseases, Jomo Kenyatta University of Agriculture and Technology, Juja, Kenya; ^2^Department of Plant and Microbial Sciences, Kenyatta University, Nairobi, Kenya; ^3^Institute of Biotechnology, Jomo Kenyatta University of Agriculture and Technology, Juja, Kenya; ^4^Center for Virus Research, Kenya Medical Research Institute, Nairobi, Kenya

In the article titled “RANTES Gene Polymorphisms Associated with HIV-1 Infections in Kenyan Population” [1], there was a missing affiliation for the first author. The corrected authors' list and affiliations are shown above.
